# Inter-organizational pooling of NICU nurses in the Dutch neonatal network: a simulation-optimization study

**DOI:** 10.1007/s10729-025-09697-8

**Published:** 2025-02-06

**Authors:** Gréanne Leeftink, Kimberley Morris, Tim Antonius, Willem de Vries, Erwin Hans

**Affiliations:** 1 Center of Healthcare Operations Improvement and Research (CHOIR), University of Twente, PO Box 217, Enschede, 7500AE The Netherlands; 2 Department of Pediatrics - Division of Neonatology Radboud UMC Amalia Children’s Hospital, Nijmegen, The Netherlands; 3 Wilhelmina Kinderziekenhuis, UMC Utrecht, Utrecht, The Netherlands; 4 Emma Kinderziekenhuis, Amsterdam UMC, Amsterdam, The Netherlands

**Keywords:** Resource sharing, Simulation-optimization, Capacity management, Neonatology, Care network design, Nurse staffing

## Abstract

Neonatology care, the care for premature and severely ill babies, is increasingly confronted with capacity challenges. The entire perinatal care chain, including the Neonatal Intensive Care Unit (NICU), operates at high occupation levels. This results in refusals, leading to undesirable transports to other centers or even abroad, which affects quality of care, length of stay, and safety of these babies, and places a heavy burden on patients, their families, and involved caregivers. In this work we assess the improvement potential of network collaboration strategies that focus on reducing the number of patient transports, by allowing flexible deployment of nurses over the existing NICUs to match short-term changes in patient demand. We develop a discrete event simulation with an integrated optimization module for shift allocation and transfer optimization. A case study for the Dutch national NICU network, involving 9 NICU locations and current transport of 15% of all NICU patients in case of no flexible deployment, shows the potential of transporting staff instead of patients: About 70% of patient transports can be eliminated in case of 15-50% capacity sharing, and about 35% of nationwide transports is eliminated with up to 15% capacity sharing in the Dutch’s main conurbation area only.

## Highlights

- Inter-organizational and adaptive resource sharing reduces patient transports
- Only limited flexibility in resource deployment is required to mitigate the largest effects of variability in patient arrivals

## Introduction

Neonatal intensive care is the care associated with severely ill or prematurely born babies, starting from 24 weeks. These patients may experience complications due to their low gestational age, weight, and developmental phase [1]. Annually, approximately 4100 newborns (2.4% of all newborns) are in need of neonatal intensive care in The Netherlands (based on data of [34] from 2012-2021). Newborns requiring intensive care and monitoring are referred to one of the nine Neonatal Intensive Care Units NICUs in The Netherlands.

**Fig. 1:**
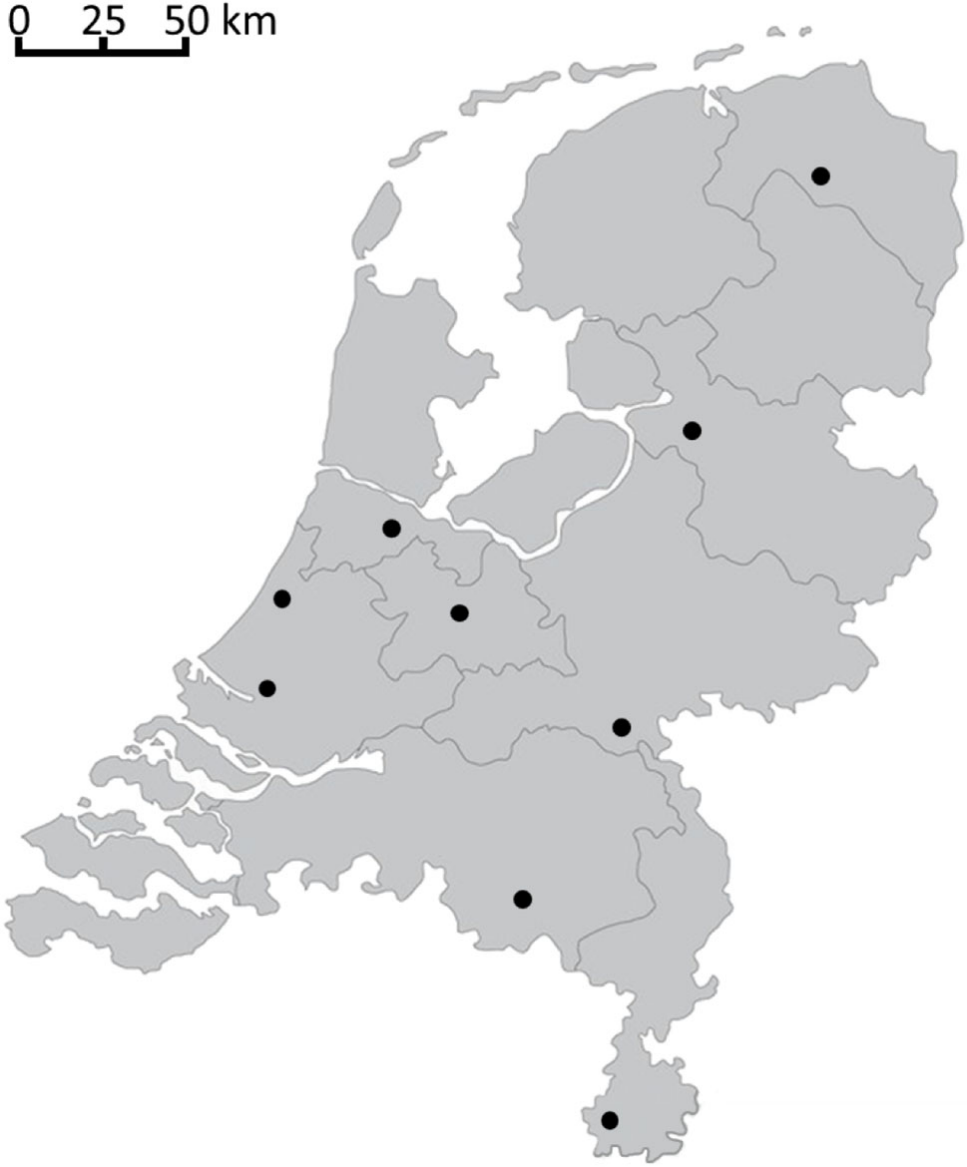
Map of The Netherlands with NICU locations

Neonatal care is an expensive care mode due to its complexity. Therefore, a trade-off has to be made between the costs of neonatal care and its accessibility. Neonatal care in The Netherlands is organized in regions and each NICU is responsible for its own catchment area, as shown in Fig. 1. Therefore, every patient is assigned a primary NICU. If a location is fully occupied, it is forced to transport a new patient from its own catchment area. This patient then has to be transported to another NICU, preferably antenatally but occasionally postnatally. In The Netherlands, the transport means have to be arranged by the primary NICU. This transport requires an ambulance, a neonatologist, and a neonatal nurse. In case the patient is transported before birth, it may also be necessary to include a gynecologist in the transport.

Currently, about 600 neonatal patient transfers (15% of all NICU admissions) occur each year [36], most of them due to full occupation of their primary NICU. Despite the deployment of a specialized transport team, transporting a patient to another NICU still comes with medical risks of transport [12, 20, 33]. Furthermore, it causes the patient to be treated by another doctor, further away from home, resulting in reduced parental engagement and increased financial strain for the involved families [7]. Therefore, there is a need to decrease the number of neonatal patient transfers. As shown in previous studies, a capacity mismatch or high targeted workload is one of the reasons for frequent patient transfers [3, 8], and should therefore be addressed first. As a second step, which is the focus of this work, we propose efficient strategies to deal with variability in patient arrivals in the nationwide neonatal network while ensuring high accessibility of neonatal care.

The capacity constraints in the NICUs mainly revolve around nursing capacity, and through that the capacity in operational beds, rather than physical bed capacity, in line with the staffing shortages prevalent in healthcare. This shortage prohibits enlarging the nursing workforce to create a capacity buffer to deal with demand variability. However, the gap between the demand and supply of nurses can also be closed by using the existing workforce more efficiently [27]. In this work, we therefore propose a flexible inter-organizational nurse collaboration approach, in which nurses are flexibly deployed based on demand realizations in the neonatal network. Instead of hiring temporary staff, a flexible nursing pool within the neonatal network could offer robustness against demand variability, while efficiently deploying personnel within the network.

The use of flexible nurses (also referred to as floating nurses, travel nurses, temporary nurses, or cross-trained nurses in this context) to cope with demand and supply variations has been shown effective as a (partial) solution to the nursing shortage problem [22, 30, 32, 40]. It reduces the need for buffer capacity [40]. Flexible staff is used in a range of departments, including highly specialized departments with an accessibility function such as intensive care units and emergency departments [13]. Flexible nurses are typically used to add flexibility to the static nurse schedules, which are usually made weeks in advance [38]. For example, it can be used as a short-term solution to filling vacancies without requiring permanent nurses to work overtime, which is expensive for hospitals [30]. Offering flexible nurses creates short-term flexibility to efficiently manage and adapt to fluctuations in demand and supply [15]. The strategy is especially useful in case of unexpected variations due to nurse absences or increased patient acuity or demand [41]. Nurses can also benefit from cross-training or floating, as it offers them more care types and variation within their job and exposes them to more challenges and learning opportunities [28]. Furthermore, [32] show that nurses who are included in temporary nursing pools have a higher retention rate, and [22] showed that using temporary staff reduces turnover and costs, and improves quality of care.

**Table 1:** Origin of patients as the percentage of all patients in 2015 (based on data of [36])

Region	% of patients from own region	% of patients from other regions
Amsterdam	91.3	8.7
Groningen	87.6	12.4
Leiden	84.0	16.0
Maastricht	91.1	8.9
Nijmegen	74.9	25.1
Rotterdam	92.4	7.6
Utrecht	76.0	24.0
Veldhoven	73.3	26.7
Zwolle	77.2	22.8
*Total*	*84.3*	*15.7*

**Table 2:** Estimated average yearly NICU demand per catchment area in the years 2015-2019 (based on data of [34])

Region	Fraction of nationwide number of births in catchment area	Estimated number of NICU admissions per year
Amsterdam	20.4	828
Groningen	9.5	385
Leiden	9.2	373
Maastricht	4.8	195
Nijmegen	8.8	357
Rotterdam	18.1	734
Utrecht	13.7	556
Veldhoven	7.7	312
Zwolle	7.7	312
*Total*	*100*	*4,052*

**Fig. 2:**
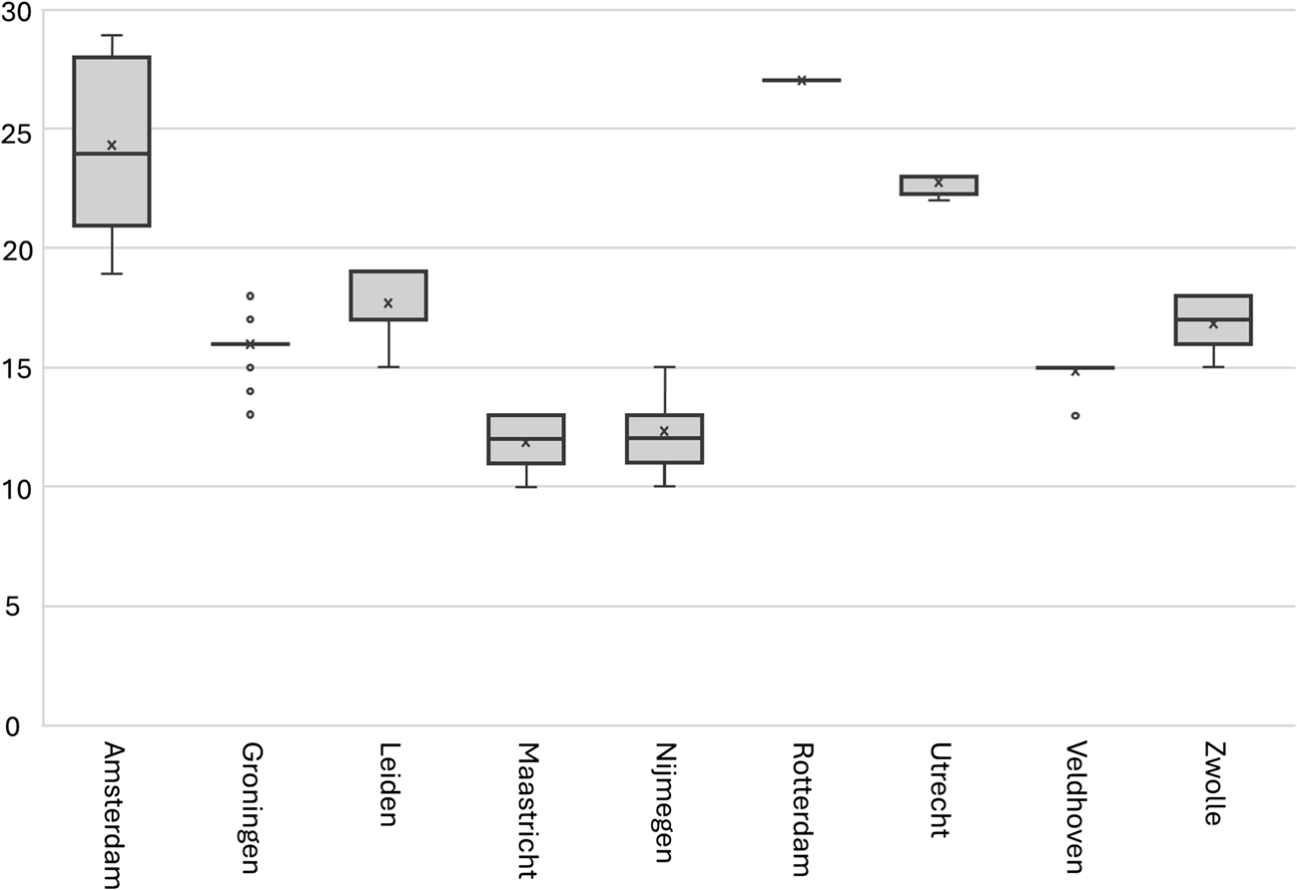
The number of operational beds at NICU locations in 2020 (based on data of BabyZoektBed)

Concluding, we hypothesize that benefits can be gained from inter-organizational adaptive nurse allocation in the Dutch NICU network. Given the modest distances between the NICUs in The Netherlands, in this paper we assess the effects on patient transport of this inter-organizational co-operative NICU nursing system that allows for the movement of patients as well as nurses between the locations, and develop a model to determine improved system settings for the Dutch neonatal network.

The remainder of this paper is as follows: Section 2 introduces the Dutch NICU network logistics and its current performance. Section 3 proposes simulation-optimization model to prospectively assess capacity sharing configurations in neonatal networks. Section 4 presents the case study of pooling NICU staff in The Netherlands, together with its results. Finally, Section 5 presents the conclusions and discussion.

## Data analysis of the Dutch NICU network logistics performance

In 2015, approximately 15% of all NICU patients in The Netherlands could not be treated at their primary NICU [36]. It is preferred to antenatally transport patients, as this poses less risk. If this is not possible, the patient is transported postnatally. Table 1 shows the percentage of patients that are from a cNICU’s own catchment area and the percentage of patients that are from other catchment areas in the year 2015. The table shows that there are some NICUs who mainly treat patients from their own region and some NICU that spend a greater amount of their capacity on patients from other catchment areas. Note that these transports both include avoidable transport, e.g., due to full occupation of the NICU, as well as transport that cannot be avoided, e.g., if a patient from a catchment area requires specialized treatment, such as pediatric cardiac surgery which is only provided in a subset of the NICU hospitals.

The Netherlands has nine NICU locations. Table 2 presents the estimated average yearly NICU demand per catchment area of these locations, based on data of [34] over 2015-2019.

**Fig. 3:**
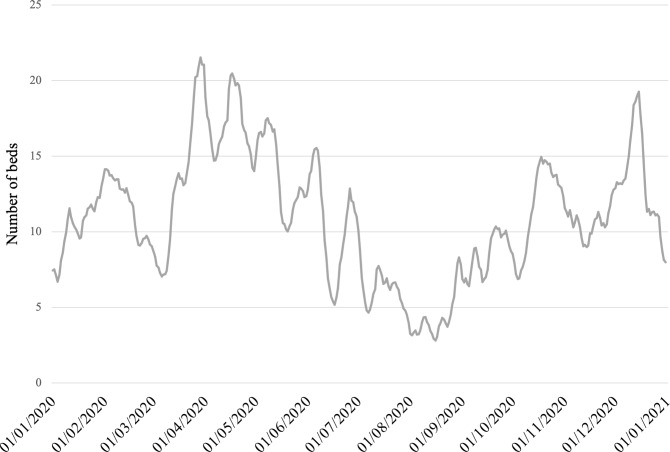
Weekly moving average of the total number of available operational NICU beds in The Netherlands in 2020 (based on data of BabyZoektBed)

The capacity, as displayed in the number of operational beds, of each NICU depends on the number of physical beds available, the number of available nurses, and the acuity of patients that have been placed at the NICU. The acuity level of a patient determines the number of NICU nurses required to care for this patient. Figure 2 shows the spread in the number of operational beds of all Dutch NICUs, based on self-reported NICU occupancy data gathered by *BabyZoekt-Bed* in 2020. The data shows that some NICUs experience more variation in the number of self-reported operational beds than others. Figure 3 shows the weekly moving average of the average number of *available operational beds* per day in 2020 of all NICUs. Although this number is very volatile, there was always at least one NICU bed available in The Netherlands in 2020. This shows that on a national level, there is enough capacity for the NICU demand in The Netherlands. However, when we look at the individual moving averages of the number of available operational beds for NICUs (not reported for privacy reasons), we see that the number of available operational beds rarely exceeds two available operational beds for most NICUs, and on average 53% of time there are no operational beds available at a NICU, with up to 82% for the most occupied NICU.

The low number of available operational beds increases the number of patient transports. As adding additional nursing capacity to the system is not a feasible option in times of staffing shortages, we need an adaptive system that is able to flexibly react to variation in patient demand and variation in operational beds in order to prevent patient transports with the current available capacity. One example of such an adaptive system is an inter-organizational flexible nursing pool [15], operating as a short-term flexible resource allocation unit, of which we will further assess improved settings and effectiveness in the remainder of this paper.

## Simulation-optimization model

To analyze the capacity deployment and patient transfers in a neonatal network, we propose a simulation-optimization model. We first discuss the relevant literature on neonatal network modeling in Section 3.1, then present some assumptions in Section 3.2. The simulation model is presented in Section 3.3, followed by the optimization model in Section 3.4. We end with the implementation in Section 3.5

### Relevant literature

Despite the clear potential and need for improving neonatal care network performance through inter-organizational collaborative approaches, the modeling of these networks has received limited attention in the literature. For single neonatal wards, several studies investigated deriving optimal nurse capacity levels using analytical, simulation, or optimization approaches [5, 17, 26]. However, the effects of cooperative capacity sharing on capacity efficiency and patient transport cannot be addressed when looking at these units in isolation.

For neonatal networks, only a few studies adopted simulation or analytical techniques for modeling the interaction between NICUs in a region. Simulation approaches are used in neonatal networks [3, 19], to support decision-making on a regional level. A Discrete Event Simulation DES approach was used to provide decision support for capacity decisions for several neonatal networks, e.g., in the UK [3] and British Columbia (Canada) [19]. Although the main goal of [19] was similar to our work, namely to evaluate the effect of capacity changes on the number of patient transfers out of their region (from Canada to the US), no flexible capacity strategies were evaluated, nor other interventions to reduce intra-regional patient transfers. However, their work shows clear potential to use DES models in evaluating the trade-offs associated with capacity changes in a healthcare network like the neonatal network. Analytical and optimization approaches are used in neonatal networks, but frequently also broader in healthcare delivery, to analyze and optimize network capacity settings and relevant characteristics of processes. Asaduzzaman and Chaussalet [4] for example used queuing models to estimate the number of required NICU beds, based on the rejection probabilities. However, as queueing models typically consider capacity a fixed resource, they are not suitable for evaluating flexible allocation of staff. Besides queueing models, integer linear programming approaches are applied for location and capacity planning [23, 24]. In these optimization models, capacity allocation decisions are made, at a fixed point in time. In the remainder of this section, we propose to combine the simulation and optimization approaches to evaluate the effect of flexible capacity sharing in neonatal networks. We will model the neonatal network using a DES, with an optimization module for capacity allocation decisions at the start of each shift using an Integer Linear Program ILP.

### Assumptions

To model the neonatal system with flexible deployment of nurses, which we refer to as *flex nurses*, several assumptions and simplifications of the Dutch neonatal network have to be made. We highlight the following:

- Each NICU works with three nurse shifts per day of eight hours, and all NICUs simultaneously change shifts.
- Nurses, including flex nurses, work at one NICU during their shift. So, a nurse starts and ends their shift at the same NICU.
- There is no difference in the type of care that NICU nurses are able to provide, and the Length-of-Stay LOS of patients is therefore independent of the NICU location or nurse that treats them.
- Travel times are independent of the home locations of patients and nurses, but are calculated from the primary NICU.
- Patients and staff are only transferred during shift changes.

Although in practice neonatal transfers happen throughout the entire day, assuming that staff and patient transfers happen during shift changes, enables us to use a systems perspective on the allocation of staff and patients, and optimally allocate the patients and staff at the same time.

### Simulation model

We here present a DES to evaluate the effect of inter-organizational collaboration in a neonatal network.

The model captures stochastic patient arrivals, acuity levels, and lengths-of-stay, for each hospital catchment area, as well as stochastic staffing capacities. For each generated patient, a LOS, primary NICU, acuity level, and required number of beds are sampled from empirical distributions. The required number of beds is included to account for twins, as we want to treat twins in the same hospital. We therefore model twins as one patient that requires two beds and where the patient acuity is multiplied by two. The operational capacity of a NICU depends on the number of beds and the availability of nurses, where we assume a fixed nurse-to-bed ratio of 1:2 (i.e., each nurse can operate two beds). Based on an empirical distribution (see Section 2), we determine the operational nursing capacity as a percentage of the maximum number of available operational beds divided by two.

**Fig. 4:**
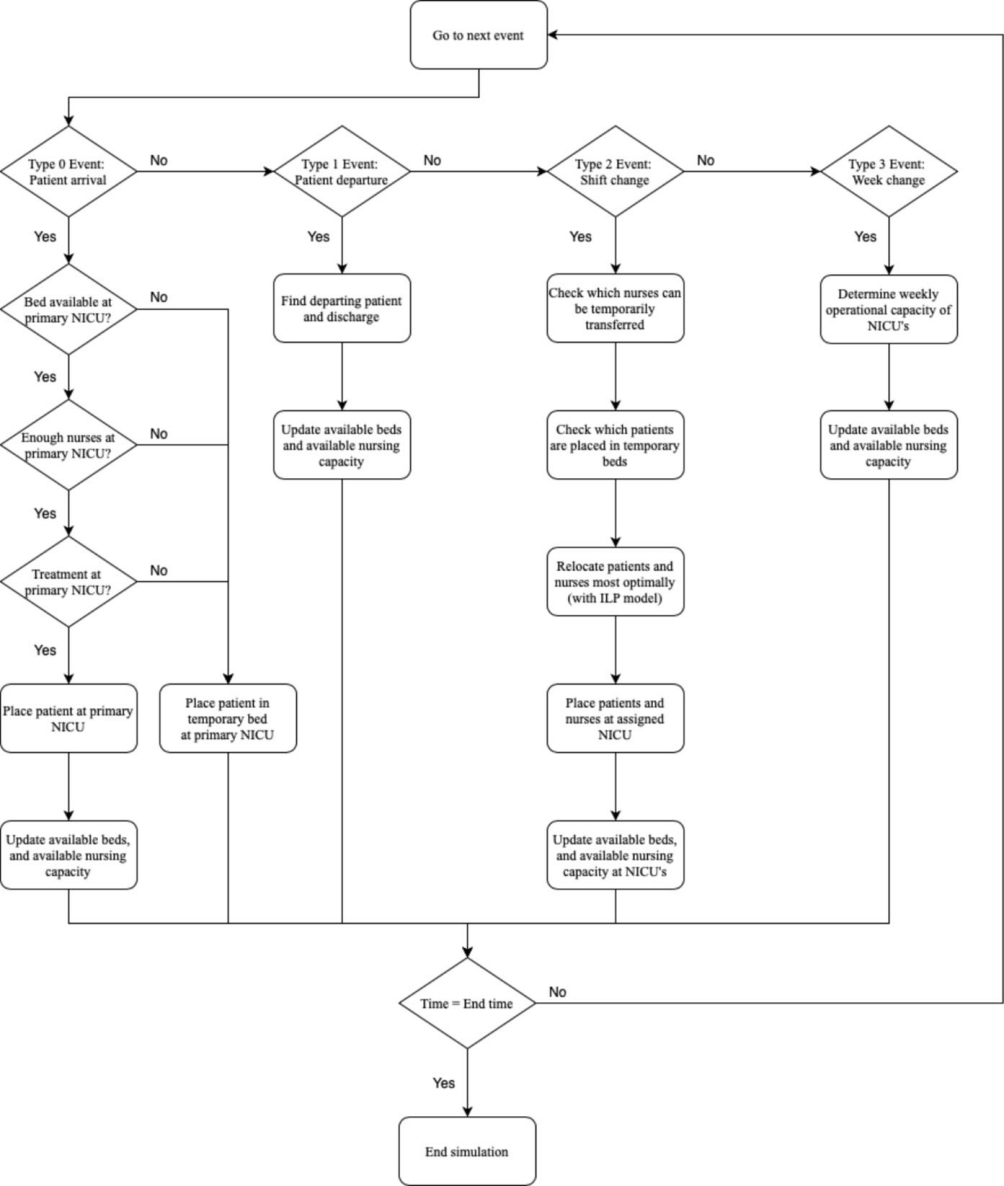
Flowchart of the DES model. There are four main event types: patient arrivals, patient departures, shift changes, and week changes. These types of events all trigger a set of actions in the model

Figure 4 shows a flowchart of the computer simulation model.

The DES model contains four different event types: patient arrival, patient departure, shift changes, and week changes. Upon arrival, patients are assigned to a bed in their primary NICU. The patient is assigned to a regular bed based on the availability of beds and the availability of nurses. The availability of beds depends on the number of non-occupied physical beds at the primary NICU. The availability of nurses depends on the cumulative acuity of the already admitted patients and the acuity level of the arriving patient, in comparison to the total nursing capacity. If no bed is available, a patient is temporarily placed in an overbed in their primary NICU. The patient will then be assigned a regular bed at the next shift change.

On *patient departure*, the patient departs from the system, which affects the number of available beds and cumulative patient acuity at the patient’s NICU.

On *week change*, the operational nursing capacity for the upcoming week is determined, to account for variation in operational capacity. If the resulting cumulative patient acuity is higher than the number of nurses present at a NICU location, this event is immediately followed by a shift change event.

On *shift change*, the patients and flexible nursing staff are optimally allocated over the NICU network. To this end, we call an ILP, as further discussed in Section 3.4, which outputs the new allocation of patients to NICUs and an assignment of where each flex nurse must work during the upcoming shift.

The simulation model measures three performance indicators:

- *Patient rejections:* the number of patients that need service abroad as a percentage of the total number of patients.
- *Patient transports:* the number of transported patients within the network as a percentage of the total number of patients.
- *Flexible nurse transfers:* the number of nurse shifts worked at a different location than the primary work location of a nurse expressed as a percentage of the total number of flex nurse shifts.

The model has been validated by comparing the model’s outputs with historical data and by discussing its working and outcomes with NICU experts. We therefore consider it to be a valid reflection of the network’s reality.

### Optimization model for nurse and patient allocation

When a shift change event occurs in the model, the flex nurses in the upcoming shift are assigned to a NICU location and the patients that are placed in temporary beds are assigned to permanent positions. An ILP is used to optimally allocate these flex nurses and patients to NICUs, given the available nurses that do not participate in the flex pool and the patients that are already placed at each NICU in regular beds. The objective of the ILP is to minimize the number of patients transported. In case a patient does need to be transported, the model relocates the patient to the nearest available NICU. Furthermore, the model takes into account the distances that flex nurses have to travel. Table 3 presents the model’s sets, parameters, and variables.

**Table 3:** The sets, parameters, and variables of the flexible nurse and patient allocation optimization model

Index and set	Definition	
$i,j \in I$	set of locations	
$p \in P$	set of patients	
Parameter	Definition	
$c_{j}$	cumulative patient acuity at location *j*	
$d_{ij}$	distance between location *i* and location *j*	
$f_{ij}$	indicator if exchange is allowed between location *i* and *j*	
$g_{p}$	acuity of patient *p*	
$h_{j}$	number of fixed nurses at location *j*	
$m_{j}$	maximum number of beds at location *j*	
$o_{j}$	number of occupied beds at location *j*	
$v_{j}$	number of flex nurses from location *j* to be allocated in upcoming shift	
$t_{p}$	number of beds required for patient *p*	
$\beta _{1} \ldots \beta _5$	weights for resp. patient traveling distance, number of transported patients, nurse traveling distance, number of nurses under-staffed, and number of rejected patients	
Variable	Definition	Range
$x_{pij}$	Indicator whether patient *p* from location *i* is treated at location *j*	binary
$z_{pi}$	Indicator whether patient *p* from location *i* is rejected (transported abroad)	binary
$y_{ij}$	Number of flex nurses of location *i* assigned to location *j* in the upcoming shift	
$b_{j}$	Number of additional staff required in location *j* (slack variable to temporarily allow under-staffing)	integer

#### Objective

1 $$\begin{aligned} \min \quad&\beta _{1} \sum _{p\in P} \sum _{i\in I} \sum _{j\in I} x_{pij} d_{ij} +\beta _{2} \sum _{p\in P} \sum _{i\in I} \sum _{j\in I} x_{pij_{i\ne j}} \nonumber \\ + \beta _{3}&\sum _{i\in I} \sum _{j\in I} y_{ij} d_{ij} + \beta _{4} \sum _{j\in I} b_{j} + \beta _{5}\sum _{p\in P} \sum _{i\in I} z_{pi} \end{aligned}$$

#### Constraints

2 $$\begin{aligned} \sum _{i\in I} \sum _{j\in I} x_{pij} + \sum _{i\in I} z_{pi}&= 1  &   \forall p\in P \end{aligned}$$

3 $$\begin{aligned} \sum _{p\in P} \sum _{i\in I} x_{pij} t_{p} + o_{j}&\le m_{j}  &   \forall j\in I \end{aligned}$$

4 $$\begin{aligned} \sum _{p\in P} \sum _{i\in I} x_{pij} g_{p} + c_{j}&\le \sum _{i\in I} y_{ij} + h_{j} + b_{j}  &   \forall j\in I \end{aligned}$$

5 $$\begin{aligned} y_{ij}&\le v_{i} f_{ij}  &   \forall i,j\in I \end{aligned}$$

6 $$\begin{aligned} \sum _{j\in I} y_{ij}&= v_{i}  &   \forall i \in I \end{aligned}$$

7 $$\begin{aligned} x_{pij}, z_{pi}&\in {0,1}  &   \forall p\in P, i,j\in I \end{aligned}$$

8 $$\begin{aligned} y_{ij}, b_j&\in \mathbb {Z}^+  &   \forall i,j\in I \end{aligned}$$

In the model, decisions must be made about the number of flex nurses with primary location *i* assigned to a location *j* ($y_{ij}$) and the location *j* where a patient is treated at the primary location *i* ($x_{pij}$). A patient who the Dutch neonatal network cannot treat, has to be rejected ($z_{pi}$). The objective function (1) is to minimize: 1. the distance traveled by patients, 2. the number of patients not treated at their primary NICU, 3. the distance traveled by all nurses, 4. nursing shortages at any location, and 5. the number of patients rejected in the network.

**Table 4:** Distance matrix NICUs expressed in kilometers

Location	Amsterdam	Groningen	Leiden	Maastricht	Nijmegen	Rotterdam	Utrecht	Veldhoven	Zwolle
Amsterdam	0	176	47	204	109	77	40	117	111
Groningen	176	0	219	337	196	250	187	261	104
Leiden	47	219	0	226	131	39	63	138	155
Maastricht	204	337	226	0	135	201	179	92	236
Nijmegen	109	196	131	135	0	116	87	75	96
Rotterdam	77	250	39	201	116	0	63	113	152
Utrecht	40	187	63	179	87	63	0	92	86
Veldhoven	117	261	138	92	75	113	92	0	160
Zwolle	111	104	155	236	96	152	86	160	0

The model is subject to several constraints. Constraints (2) ensure every patient is assigned to their primary NICU, another NICU location, or treated abroad. Constraints (3) ensure that the number of assigned patients to a location does not exceed the number of available beds. Note that due to the specific way of modeling twins, the number of patients that are assigned to a NICU is multiplied by the number of beds required for a patient. Constraints (4) ensure whether a NICU location has sufficient nursing capacity to admit an additional patient in a regular bed, by relating the cumulative acuity of assigned patients to the number of fixed and flexible nurses assigned to this location. We introduce the slack variable $b_j$ to allow temporary understaffing at a NICU *j*. Note that one should set the penalty for accepting understaffing sufficiently higher than the penalty for transporting or rejecting a patient, so the model will not assign patients to this location if it already has a nursing shortage, unless extra flexible nurses are assigned. Constraints (5) allow no nurse exchanges between locations that are not allowed to share nurses. Constraints (6) limit the capacity that each location offers to the flex pool.

### Implementation

The simulation-optimization model is implemented in Python 3.7, using NumPy and MIP Python libraries with Gurobi solver. Steady-state simulations are run for 10 years with 10 replications. A warm-up period of 1 year is used to ensure a steady state is reached before collection of outcomes.

## Experiment settings and results

This section introduces the Dutch neonatal network instance (Section 4.1), the experiment settings (Section 4.2), and the results (Section 4.3).

### Instance settings

We consider the Dutch neonatal care network, with 9 NICU locations. Table 4 shows the *distances* between NICUs expressed in kilometers.

The patient characteristics are as follows: We assume *patient arrivals* according to a Poisson distribution with exponential arrival times, with $\lambda$ the average number of births in The Netherlands. Patients are assigned to one of the NICU’s catchment areas based on the fraction of births in each catchment area (see Table 2). The *LOS* follows an exponential distribution with $\lambda$=11 days, as derived from historical data, and in line with recent literature on neonatal LOS predictions [25]. The patient *acuity level* is sampled from an empirical distribution as derived from [21] (see Table 5). In The Netherlands, there is a 1.6% probability that a pregnancy results in multiple births, with the vast majority being twins. Therefore, with respect to the* required number of beds*, each patient has a 1.6% probability of being a twin in need of two beds instead of one.

**Table 5:** Acuity distribution of NICU patients expressed as the number of NICU nurses required to care for one patient with a certain acuity level [21]

Level	Probability	Nurse per patient
1	0.00	0.20
2	0.04	0.25
3	0.39	0.33
4	0.25	0.50
5	0.25	0.67
6	0.07	1.00

The *operational capacity* for each week is based on an empirical distribution derived from the self-reported operational capacity data for the Dutch NICUs over a period of one year (2020). Table 6 shows the rounded occurrence of a certain level of operational capacity expressed as a percentage of the maximum bed capacity. For example, for 50% of the time, a NICU can only operate 80% of its maximum bed capacity.

**Table 6:** Estimation of the distribution of the level of operational capacity at NICU locations, source: WKZ

Percentage of maximum capacity (%)	Occurrence (%)
60	5
70	5
80	50
90	10
100	30

The parameter input for the *optimization model for nurse and patient allocation* is derived from the current state of the simulation model, as well as the experiment settings as discussed next in Section 4.2 (e.g., exchange indicator and size of the flexible nursing pool). The weight settings of this multi-objective optimization model are determined in such a way that we find the Pareto optimal solution that minimizes nursing shortages in any location, as this is the main compromiser of quality of care. Within the remaining set of feasible solutions, we minimize the number of patients that cannot be served in the system. Then, we minimize the number of patients not treated at their primary NICU. Finally, we minimize the number of kilometers travelled. We scaled the weights to $\beta _1$, leading to $\beta _1$ = 1, $\beta _2$ = 1000, $\beta _3$ = 1, $\beta _4$ = 5000000, $\beta _5$ = 50000.

**Fig. 5:**
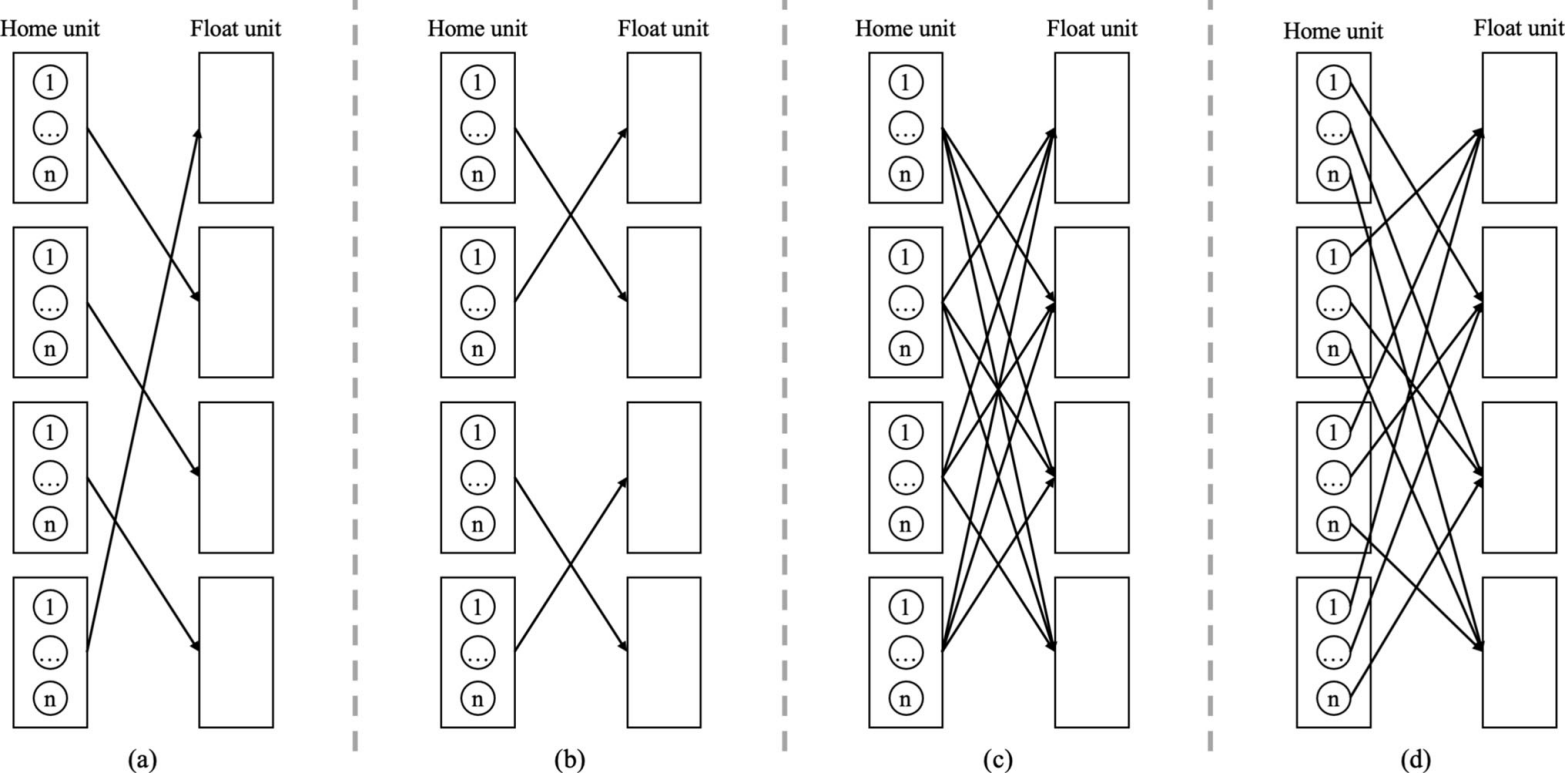
Graphical representation of cross-training policies where (a) represents the chaining policy, (b) the reciprocal pairs policy, (c) the N-to-all policy, and (d) the one-for-each policy (adapted from [18])

**Fig. 6:**
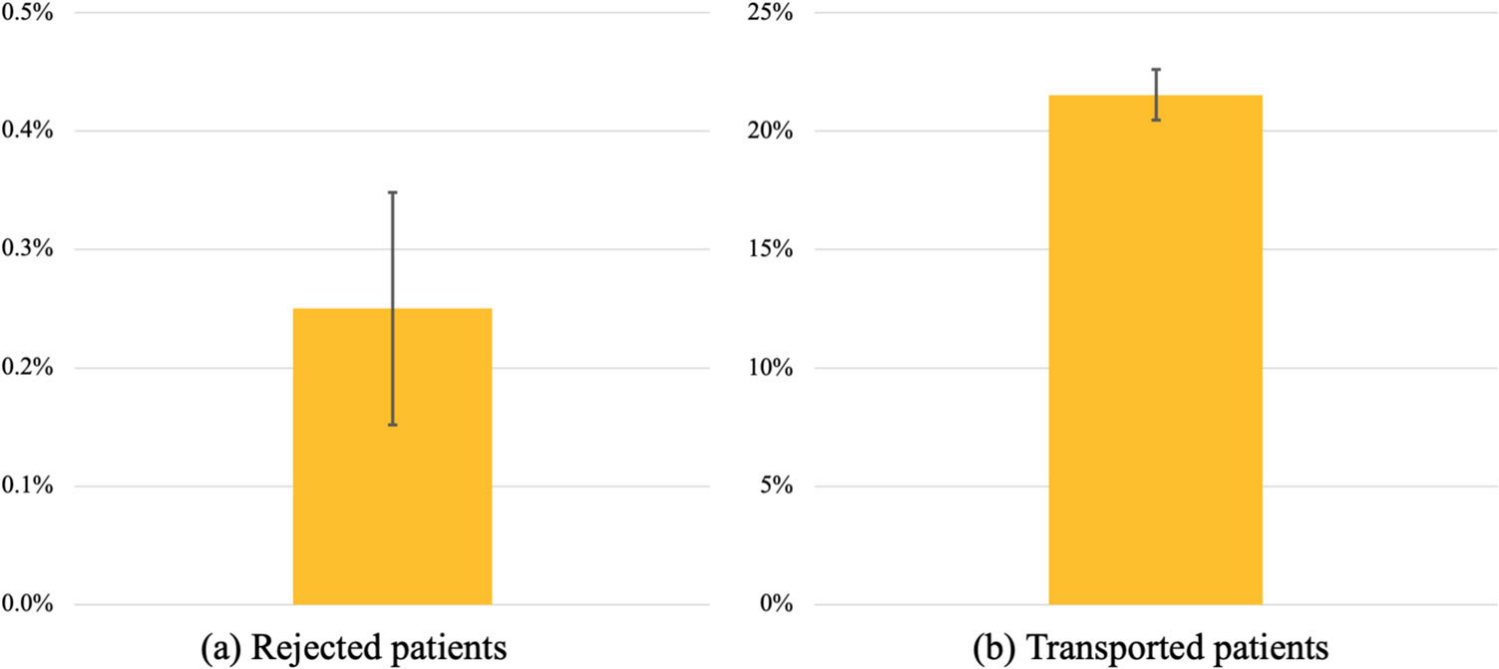
Baseline measurement of (a) the number of rejected patients as a percentage of the total number of patients and (b) the number of transported patients as a percentage of the total number of patients. The error bars represent the 95%-CI of the true mean of the key performance indicator

### Experiments

The model allows to prospectively assess various flex pool settings. We consider three factors: the number of participating nurses in the flex pool per location, the actual NICUs that participate in the flex pool, and the cross-training policy considered.

For *the number of participating nurses in the flex pool*, we consider three settings: one participating nurse per location, two participating nurses per location, and five participating nurses per location. Note that we implemented these settings in line with the national guidelines, that state that each NICU must always have at least ten to twelve staffed operational beds. This means that despite the setting, solutions are restricted to always have a minimum of five nurses remaining at the primary NICU.

For *the NICUs that participate in the flex pool*, we consider three settings: all locations, all locations except Groningen and Maastricht (as they are the furthest away), and only including the four locations in the so-called *Randstad* region, the most densely populated area of the country (as they are geographically centralized).

**Fig. 7:**
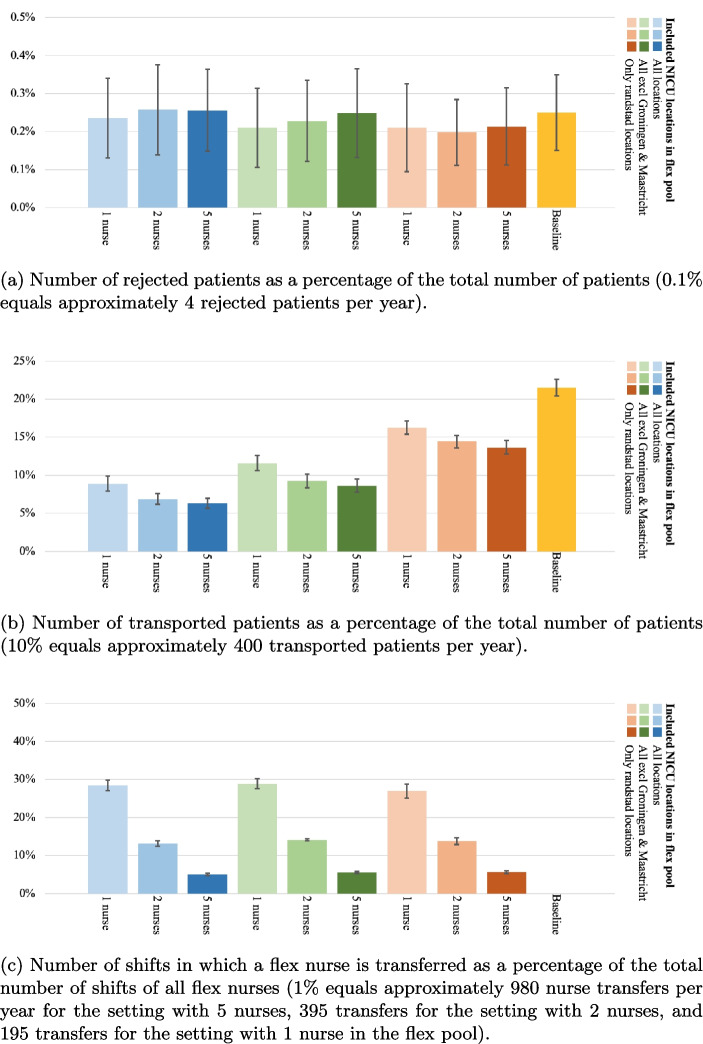
N-to-all cross-training policy experiment results

For *the cross-training policies*, we consider the N-to-all, reciprocal pairs, and chaining policies. Fügener et al. [18] describe different cross-training policies, including chaining, reciprocal pairs, N-to-all, and one-for-each, as visualized in Fig. 5. They describe the policy of chaining (Fig. 5a) as a situation where every unit trains nurses for one other location. In this way, every unit can be supplied with additional nurses from a different unit. In the cross-training policy reciprocal pairs (Fig. 5b), units are linked to each other and are able to exchange nurses between the locations. Figure 5c shows the N-to-all policy, in which units train nurses to work at all other locations. Finally, the policy one-for-each (Fig. 5d) is a policy in which units train nurses for only one other location, but where combined nurses are trained for all other locations. Due to the complexity of this system, we do not consider this policy in the experiments.

The instance settings for the chaining and reciprocal pairs cross-training policies are determined by modeling the instances as a traveling salesman problem and a capacitated vehicle routing problem respectively. These models, as well as the instance settings for the Dutch neonatal network instance, are presented in Appendix B.

Through a full-factorial experiment design, the number of experiments amounts to 27 experiments. In addition, a baseline measurement is run with zero participating nurses in the flex pool, summing to 28 experiments in total.

### Results

First, we will present a baseline measurement of the simulation model without using the flex nursing pool in Section 4.3.1. Then, in Sections 4.3.2, 4.3.3, and 4.3.4 we will present the results of the experiments that are performed for each cross-training policy. Section 4.3.5 will compare the outcomes of the different cross-training policies.

#### Baseline measurement

Figure 6 presents the outcomes of the baseline setting, without inter-organizational sharing of nurses. The percentage of rejected patients is 0.25% on average (95-CI={0.15%, 0.35%}), which means on a yearly basis 6 to 14 patients are transferred abroad. The number of transported patients as a percentage of the total number of patients is 21.53% on average (95-CI={20.46%, 22.60%}), which corresponds to approximately 840 to 920 patient transfers per year. Note that this number is higher than the reported transfers from practice, which can be due to NICUs’ capabilities to mitigate capacity shortages in the short term, e.g., through expediting the discharge of another patient to a Medium Care Unit, for the benefit of a reduction of patient transfers.

#### N-to-all cross-training policy

Figure 7a shows the number of rejected patients as a percentage of the total number of patients for all N-to-all cross-training policy experiments as well as the baseline measurement. In these experiments, the number of participating nurses in the flex pool is varied, as shown on the horizontal axis. The different colors in the figure represent the various configurations regarding the number of participating locations. The number of rejected patients is relatively stable when introducing various configurations of the flex pool, with all CIs between 0.09% and 0.38% (resp. 4 to 15 patients per year). This result is unsurprising, as the network’s overall nursing capacity is not increased or decreased when introducing a flex pool.

Introducing a flex pool with the N-to-all policy significantly reduces the number of patient transports, as shown in Fig. 7b. The best performing experiment with the N-to-all policy (*all locations - 5 nurses*) reduces the patient transports by approximately 70% (mean=6.31%, 95-CI={5.63%, 6.98%}, which corresponds to 230 to 280 patient transports per year). The worst performing experiment with the N-to-all policy (*4 locations - 1 nurse*) still reduces the patient transports by approximately 25% compared to the baseline measurement (mean=16.26%, 95-CI={15.39%, 17.13%}). The number of transported patients significantly increases as the number of participating locations decreases. Furthermore, a significant decrease in transports is observed if the number of participating nurses increases from one nurse to two nurses. However, further increasing the number of flexible nurses per location from two to five does not make a significant difference. Increasing the number of participating nurses results in more capacity that can be moved when demand requires it, which explains why a reduction in patient transports is observed. However, this effect has an asymptotic behavior, due to the maximum number of beds at NICU locations and the occupancy of the system. When NICUs are fully occupied, patients must be transported regardless of the availability of flex nurses.

**Fig. 8:**
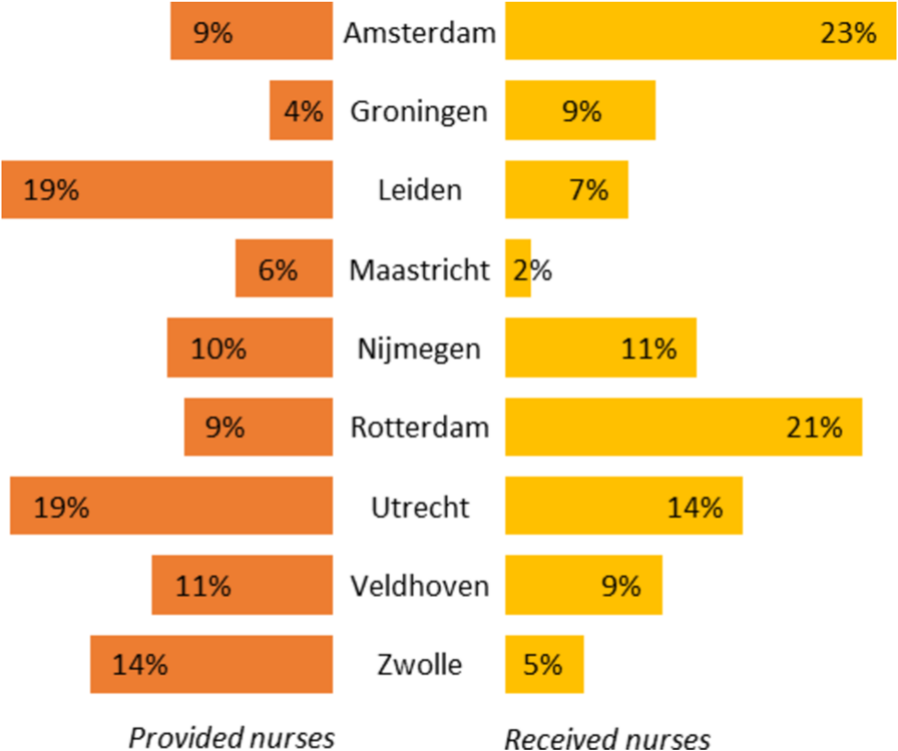
The exchanges between NICU locations in an experiment with five participating nurses, all locations included, and the N-to-all policy. Left the number of *provided* flex nurses at each location as a percentage of the total nurse exchanges, and right the number of *received* flex nurses at each NICU location as a percentage of the total nurse exchanges

Figure 7c shows the number of nurse transfers as a percentage of the total number of shifts of all flex nurses for the N-to-all cross-training policy experiments. The percentage significantly decreases as the number of nurses increases, from 28%, to 14%, to 5% for 1, 2, and 5 nurses respectively, which is as expected as the transfers can be divided among more nurses. The number of participating locations does not significantly influence the percentage of nurse exchanges per participating flex nurse.

Figure 8 gives an overview of the number of times NICUs have received or provided a flex nurse as a percentage of the total number of exchanges in the model for the best performing experiment with the N-to-all policy (*all locations - 5 nurses*). This shows that the flex pool is mainly used by larger NICU locations and locations that are centrally located (e.g., the three largest NICUs: Amsterdam (22.79% received), Rotterdam (22.85% received), and Utrecht (13.88% received)). Locations that are less central, such as Groningen and Maastricht, only receive and provide a limited number of nurses.

#### Reciprocal pairs cross-training policy

**Fig. 9:**
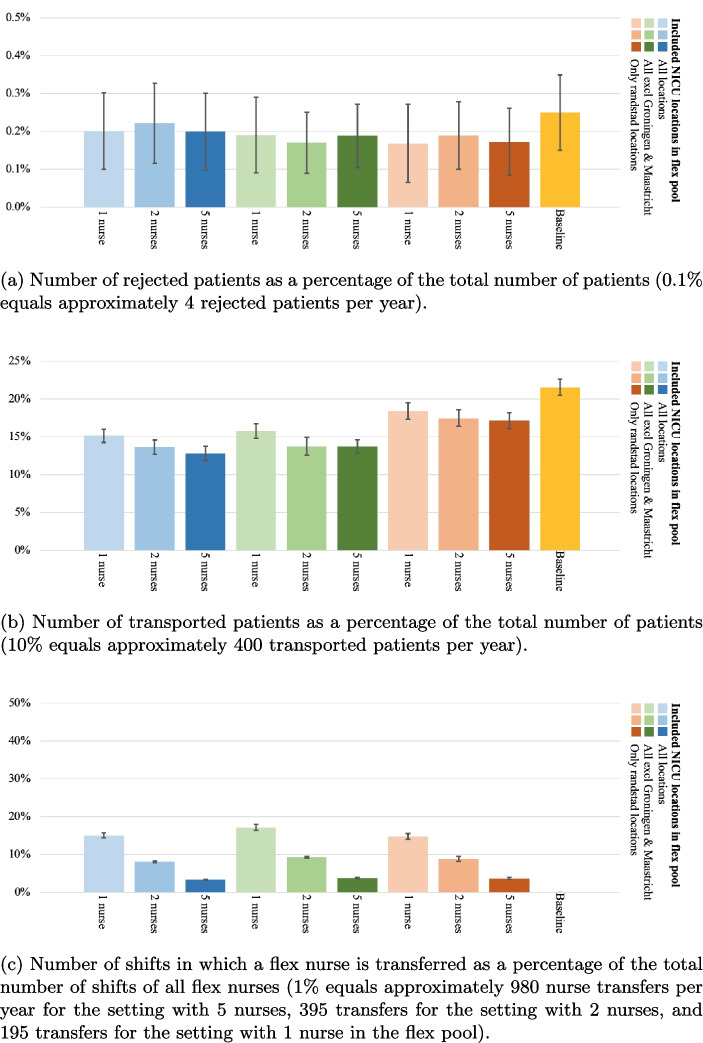
Reciprocal pairs cross-training policy experiment results

The number of rejected patients is again relatively stable when various configurations of the flex pool are introduced in the reciprocal pairs cross-training policy, with all CIs between 0.06% and 0.33% (resp. 3 to 14 patients per year), see Fig. 9a. This is slightly lower than the baseline measurement, but not significant.

Introducing a flex pool with the reciprocal pairs policy significantly reduces the number of patient transports by up to 35%, as shown in Fig. 9b. The results for the configurations with all locations (blue, 5 nurses - 95-CI={11.8%, 13.8%}) and the configurations with all locations except Groningen and Maastricht (green, 5 nurses - 95-CI={12.8%, 14.6%}) are comparable, while the setting with only Randstad locations (orange, 5 nurses - 95-CI={16.0%, 18.2%}) performs slightly worse.

Figure 9c shows the number of nurse transfers as a percentage of the total number of shifts of all flex nurses for the reciprocal pairs cross-training policy experiments, which shows similar outcome behavior as the N-to-all policy. The percentage significantly decreases as the number of nurses increases, from 16%, to 9%, to 4% for 1, 2, and 5 nurses respectively. This shows the intuitive consequence that having less people in the flex pool increases the probability of being flexibly deployed for the nurses in the flex pool. Note that the number of participating locations does not significantly influence the percentage of nurse exchanges per participating flex nurse.

#### Chaining cross-training policy

**Fig. 10:**
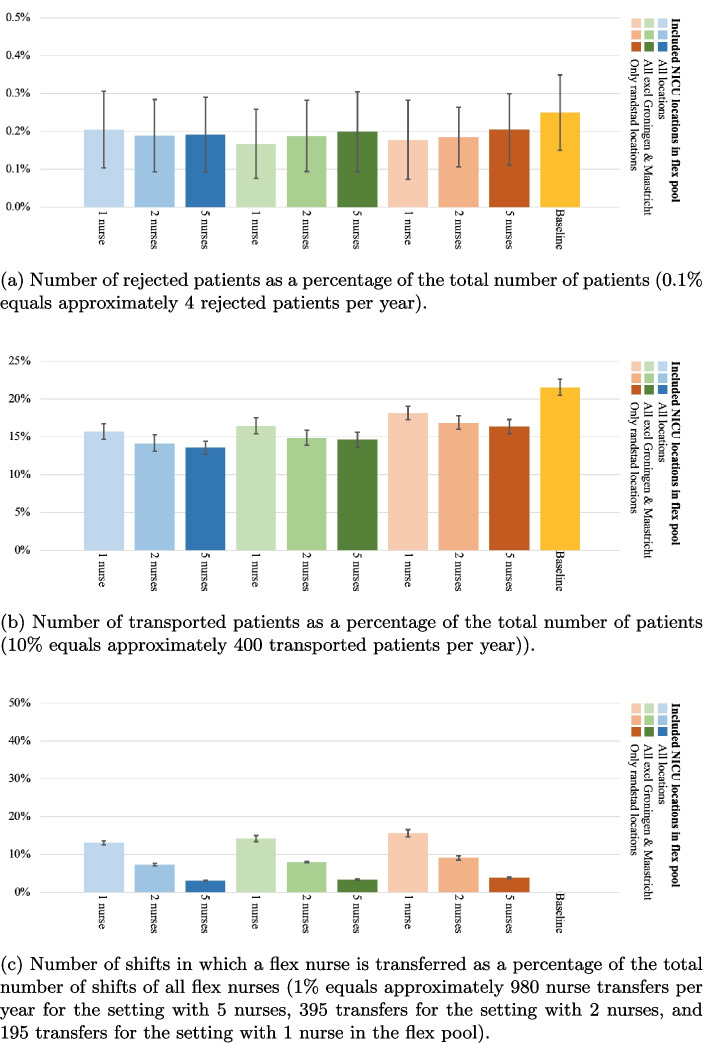
Chaining cross-training policy experiment results

The number of rejected patients is again relatively stable when various configurations of the flex pool are introduced in the chaining cross-training policy, with all CIs between 0.07% and 0.35% (resp. 3 to 14 patients per year), see Fig. 10a, similar to the baseline measurement.

Introducing a flex pool with the chaining policy significantly reduces the number of patient transports by up to 35%, as shown in Fig. 10b. Again, the results for the configurations with all locations (blue, 5 nurses - 95-CI={12.7%, 14.4%}) and the configurations with all locations except Groningen and Maastricht (green, 5 nurses - 95-CI={13.9%, 15.6%}) are comparable. However, the difference between these two configurations and the configurations in which only Randstad locations are included (orange, 5 nurses - 95-CI={15.4%, 17.3%}) is smaller than in other cross-training policies.

Figure 10c shows the number of nurse transfers as a percentage of the total number of shifts of all flex nurses for the chaining cross-training policy experiments, which shows similar outcome behavior as the other cross-training policies. The percentage significantly decreases as the number of nurses increases, from 14%, to 8%, to 3% for 1, 2, and 5 nurses respectively. The number of participating locations does not significantly influence the percentage of nurse exchanges per participating flex nurse.

#### Comparison of cross-training policies

Comparing the effects of the different cross-training policies reveals several managerial insights. First, as expected, the N-to-all cross-training policy provides the best performance in the number of patient transports as a percentage of the total patients for each identified experiment. Also, having more locations in the flex pool improves patient transport performance. However, for the reciprocal pairs policy, we see only a slight, but not significant, decrease in the configurations where all locations are incorporated and where all locations except Groningen and Maastricht are incorporated. The reason for this stagnated improvement is subject for further research.

Second, to obtain the large reduction in patient transports, the N-to-all policy requires the most nurse transfers for each experiment, almost doubling the number of nurse transfers compared to the other cross-training policies. This increase in nurse transfers is expected, as the other policies only allow a limited number of locations to exchange nurses with. Note that for the flex nurses in any of these policies, even though they can be flexibly deployed in another NICU, they still work the majority of time (72% - 97% on average, depending on the setting) in their primary NICU.

Third, there are slight differences between the percentage of rejected patients in the various cross-training policies and number of flex nurses, but these differences are not significant. Independent of the settings, the whole neonatal network still functions as one large capacity pool for transfers outside the network.

## Conclusion and discussion

A large number of neonatal transfers in the Dutch neonatal system are due to nurse capacity challenges. Our simulation-optimization approach, with an integrated flexible staff optimization module in the DES environment, models a network of NICUs and allows analysis of the impact of various possible interventions and scenarios, such as capacity changes and various flexible staffing policies.

In this work, we showed that the implementation of an inter-organizational nursing flex pool significantly reduces patient transport, as the number of participating nurses and number of participating locations are both negatively correlated with the number of patient transports. We showed how efficient use of neonatal staff by sharing 5 nurses per NICU location in the entire Dutch neonatal network will reduce the number of patient transports by 70% (610-640 less patient transports per year) while at the same time efficiently deploying the scarce nursing capacity. A more realistic setting, where the capacity sharing of 2 nurses per NICU location is only allowed between neighboring NICUs, without the two outlier locations participating in the pool, will reduce nationwide patient transports by 35%.

Multiple studies in various countries found correlations between neonatal transport in the first 48 hours of life and severe health outcomes, such as higher odds of severe brain injury (1.19 to 1.60 [20]), and particularly increased incidence and severity of intraventricular haemorrhages (27.4% compared to 13.4% for non-transported neonatal infants, of which 44.1% compared to 32.9% was severe [33]). Extrapolating these health outcomes to our best-case setting, with a 70% reduction in neonatal transports compared to the current situation, these numbers indicate that in The Netherlands on a yearly basis intraventricular haemorrhages in about 85-89 preterm infants, of which 47-49 suffering from severe intraventricular haemorrhages, can be prevented by inter-organizational pooling of NICU nurses.

Reducing patient transports is attained through flexible deployment of nurses. Our study showed that to reduce one patient transport, one flexible nurse has to work approximately four shifts at another NICU location. This 1:4 ratio is caused by the fact that for each reduced patient transport, that patient stays in its primary NICU for the entire duration of the hospital admission. So not only a reduction in travel for the patient and the accompanying team is realized, but also continuity of care increases. However, this system in which the right patient is treated at the right place, comes at a cost: Hospitals may face an increase in staffing costs (due to flexible deployment) and CO2-emissions will increase due to increased traveling.

Setting up an inter-organizational collaboration with staff sharing in healthcare is challenging. However, efficient deployment of personnel has proven to be the most important efficiency reason for setting up an inter-organizational collaboration [43]. Our work provides evidence of these efficiency gains, thereby opening the debate with strong motives for such an efficiency-driven collaboration. Further analyses could focus on the economic evaluation of this intervention, to also provide a cost perspective, both on the system level as well as on the level of the individual healthcare provider. There are no significant performance differences between the various cross-training strategies. In the N-to-all policy, all NICUs exchange nurses with each other, in the reciprocal pairs policy a NICU exchanges nurses with one other NICU and in the chaining policy the NICUs form a chain and receive nurses from a different location than the location they send nurses to. Given the limited effect on the performance measures, the reciprocal pairs policy might be preferred as a collaborative strategy, as this has the lowest geographical burden, could lead to more familiarity between nurses of the collaborating units and therefore increase the community feeling, could improve collaboration between their organizations, and requires nurses to get familiar with just one other unit’s way of working [16].

To ensure adequate quality of care when using flexible nurses, they must be trained to work in different environments, which can be a difficult process [9]. To this end, the reciprocal pairs and chaining cross-training policies already limit the number of different environments for nurses to work in to one or two non-primary NICUs, and are therefore promising cross-training policies for implementation in the inter-organizational collaboration. When flexible nurses are assigned to a new unit, they should have time for an onboarding or orientation process before starting their first shift in this new environment [37, 42]. One way to ensure that nurses are capable and qualified to work in a non-primary unit is by pairing them with nurses who have a permanent position in this unit [29]. This allows the flexible nurses to be trained and to adjust to patient needs at non-primary locations.

Working in multiple locations means that nurses must be familiar with the way of working and procedures of multiple units. This is why some literature suggests that there are potential safety issues with the use of flexible staff [39, 40]. For example, [35] argues that flexible nurses cause more severe medication errors than permanent staff. On the other hand, [6] found that there is no evidence that links flexible staff to an increased risk of infections. Aiken et al. [2] conclude that flexible nurses are not less qualified or competent to work in the departments they have been trained for than permanent staff at these locations and that they do not diminish the quality of care provided. What literature does agree on, however, is that using flexible nurses is only safe when they are sufficiently experienced. As familiarizing oneself with the procedures of multiple hospitals is rather complex, some hospitals discourage newly graduated nurses from joining float pools and prefer to include more experienced nurses [10].

Another challenge for implementation is the notification of flexible staff regarding their unit of work at the start of each staff. Currently, we assumed staff is informed right before the start of their shift, without any compensation for traveling to potential units further away. Ensuring that nurses would know at home where to travel to for work, is one of the main reasons to consider the Randstad configuration, as we can assume that average travel times to all hospitals in this region are somewhat similar for nurses in a hospital in that region.

In our simulation-optimization model we made several assumptions that deviate from reality. For example, in the model, patients are only transferred during shift changes, whereas in reality patients are usually antenatally transported to the NICU that will treat them at any time of the day. However, as nurses can only be reassigned at the start of a shift, and since we want to use the flexibility that a system’s perspective considering all patients provides, we set the decision moments equal to the moments of shift change. Future research should analyze the impact of online (on-arrival) patient-to-NICU assignment on the neonatal care system, and identify well-performing online patient allocation strategies.

## Figures and Tables

**Fig. 11:**
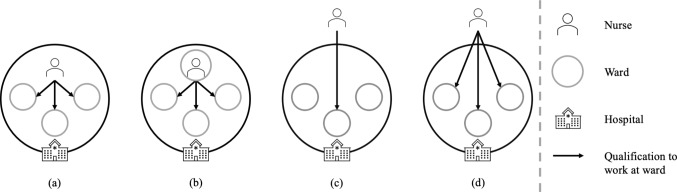
Graphical representation of temporary nurse types where a) represents float nurses, b) cross-trained nurses, c) resource team nurses, and d) agency nurses

**Fig. 12:**
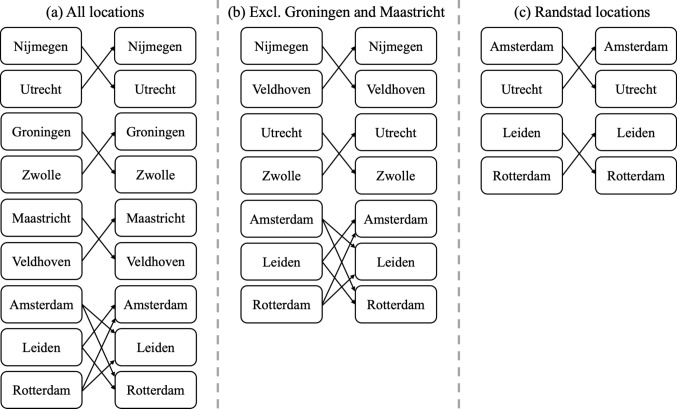
Allowed nurse exchanges in the reciprocal pairs cross-training policy determined by a capacitated vehicle routing problem that minimizes total travel distance

**Fig. 13:**
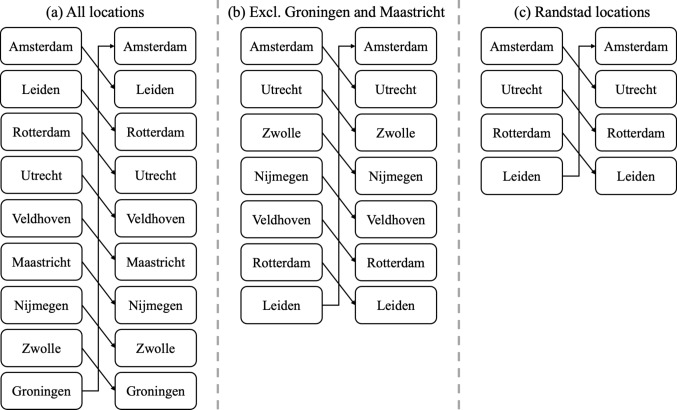
Allowed nurse exchanges in the chaining cross-training policy determined by a traveling salesman problem that minimizes total travel distance

## Data Availability

Data is available with the authors upon request. Code is available with the authors upon request.

## Appendix A: Temporary staffing configurations

We distinguish four temporary staffing configurations, as graphically represented in Fig. 11. *Float pools*, as shown in Fig. 11a, refer to systems in which nurses are employed by a hospital and trained to work at different departments [11]. In this system, nurses are not dedicated to a specific unit but instead are assigned to understaffed wards at the beginning of their shift. *Cross-trained nurses* (see Figure 11b) are similar to float nurses, however, they are primarily trained for one unit and may temporarily be placed at other departments only when demand requires it [18]. *Resource teams* (see Fig. 11c) include nurses that are not affiliated with a specific hospital but instead are employed directly by the resource team [14]. The benefit of resource teams is that the nurses included are organized by clinical expertise. *Temporary nursing agencies* (see Fig. 11d) are similar to resource teams. [31] define agency nurses as nurses who are employed by an external agency and are temporarily deployed at hospitals as additional staffing. So, in contrast to nurses from resource teams, these nurses do not have a certain expertise and are capable of working in different departments. In this paper, we focus on effective deployment of cross-trained nurses.

## Appendix B: Cross-training policies

This appendix describes the models to determine the instance settings for the reciprocal pairs cross-training policy in Section 7.1 and the chaining cross-training policy in Section 7.2. It also presents the outcomes of these models for the instance of the Dutch neonatal network.

### B.1 Reciprocal pairs policy

In the reciprocal pairs policy, locations are paired and only share nurses between each pair. In this way, floating between NICUs is possible whilst limiting the number of locations nurses have to be familiar with to only two. The pairs are made by ensuring the least amount of cumulative travel time for nurses and calculated for each combination of included locations in the experiments. This means that the pairs are made for the situation where all locations are included, where all locations are included except Groningen and Maastricht, and where only the centrally located NICUs are included.

The reciprocal pairs policy is modeled as a capacitated vehicle routing problem (CVRP). Every NICU location in the network is a location with a demand of one. The NICU locations also represent the depots. The number of vehicles in the CVRP is equal to the number of locations divided by two and their capacities are equal to two. In case of an odd number of locations, the number of vehicles equals the number of locations divided by two rounded down. The capacities of the vehicles are equal to two and one vehicle has a capacity of three. In this way, the model finds the shortest routes between all locations and identifies the shortest cumulative distance for the reciprocal pairs policy.

**Table 7:** Notation reciprocal pairs policy CVRP model

Sets	
$h,i,j \in \mathcal {V}$	set of all locations and depots, $\mathcal {V}= \{1, \ldots , V\}$
$h,i,j \in \mathcal {I}$	set of all locations, $\mathcal {I}= \{1, \ldots , I\}$, with $\mathcal {I} \subset \mathcal {V}$
$h,i,j \in \mathcal {D}$	set of all depots, $\mathcal {D}= \{1, \ldots , D\}$, with $\mathcal {D} \subset \mathcal {V}$
$k \in \mathcal {K}$	set of all vehicles, $\mathcal {K}= \{1, \ldots , K\}$
Parameters	
$d_{ij}$	distance between location/depot *i* and location/depot *j*
$q_{i}$	demand of location *i*
$p_{k}$	capacity of vehicle *k*
Decision variables	
$x_{ijk}$	binary indicator that equals 1 if vehicle *k* is used for arc (*i*, *j*), 0 otherwise

We first introduce some notation in Table 7.

#### Objective

B1 $$\begin{aligned} \min \quad&\sum _{i\in \mathcal {V}} \sum _{j\in \mathcal {V}} \sum _{k\in \mathcal {K}} d_{ij}x_{ijk} \end{aligned}$$

#### Constraints

B2 $$\begin{aligned} \sum _{i\in \mathcal {V}} \sum _{k\in \mathcal {K}} x_{ijk}&= 1  &   \forall j\in \mathcal {I} \end{aligned}$$

B3 $$\begin{aligned} \sum _{j\in \mathcal {V}} \sum _{k\in \mathcal {K}} x_{ijk}&= 1  &   \forall i\in \mathcal {I} \end{aligned}$$

B4 $$\begin{aligned} \sum _{i\in \mathcal {V}} x_{ihk} - \sum _{j\in V} x_{hjk}&= 1  &   \forall k\in \mathcal {K}, h\in \mathcal {I} \end{aligned}$$

B5 $$\begin{aligned} \sum _{i\in \mathcal {D}} \sum _{j\in \mathcal {I}} x_{ijk}&\le 1  &   \forall k\in \mathcal {K} \end{aligned}$$

B6 $$\begin{aligned} \sum _{j\in \mathcal {D}} \sum _{i\in \mathcal {I}} x_{ijk}&\le 1  &   \forall k\in \mathcal {K} \end{aligned}$$

B7 $$\begin{aligned} \sum _{i\in \mathcal {I}} q_{i} \sum _{j\in \mathcal {V}} x_{ijk}&\le p_{k}  &   \forall k\in \mathcal {K} \end{aligned}$$

B8 $$\begin{aligned} x_{ijk}&\in \{0,1\}  &   \forall i,j\in \mathcal {V}, k\in \mathcal {K} \end{aligned}$$

The CVRP’s objective is to minimize the distance between paired locations. Constraints (B2) and (B3) ensure that all locations are visited and constraint (B4) ensures that a vehicles that visits a location also leaves that location. Constraints (B5) and (B6) ensure vehicles depart and arrive at most once from and to each depot. The capacity of the vehicles is managed by constraint (B7).

The identified pairs for the Dutch neonatal network are shown in Fig. 12. Because the NICU network in The Netherlands has an odd number of locations, the choice was made to add the remaining location to one of the pairs. This is done for experiments in which all locations are included (Fig. 12a) and experiments for which all locations are included except for Groningen and Maastricht (Fig. 12b).

### B.2 Chaining policy

The chaining policy allows each NICU location to send nurses to one flex location and allows each NICU location to receive nurses from one flex location. The chain is determined by creating the shortest route possible between the NICUs included in the flex pool and this is calculated by modeling the system as a traveling salesman problem (TSP).

We first introduce some notation in Table 8.

**Table 8:** Notation chaining policy TSP model

Sets	
$i \in \mathcal {I}$	set of all locations, $\mathcal {I}= \{1, \ldots , I\}$
$s \in \mathcal {S}$	set of all sub-tours, $\mathcal {S} = \{1, \ldots , S\}$
Parameters	
$d_{ij}$	distance between location *i* and location *j*
Decision variables	
$x_{ij}$	binary indicator that equals 1 if arc (*i*, *j*) is in the tour, 0 otherwise

#### Objective

B1 $$\begin{aligned} \min \quad&\sum _{i\in \mathcal {I}} \sum _{j\in \mathcal {I}} d_{ij}x_{ij} \end{aligned}$$

#### Constraints

B2 $$\begin{aligned} \sum _{i\in \mathcal {I}} x_{ij}&= 1  &   \forall j\in \mathcal {I} \end{aligned}$$

B3 $$\begin{aligned} \sum _{j\in \mathcal {I}} x_{ij}&= 1  &   \forall i\in \mathcal {I} \end{aligned}$$

B4 $$\begin{aligned} \sum _{i,j\in \mathcal {S}} x_{ij}&\le S-1  &   \end{aligned}$$

B5 $$\begin{aligned} x_{ij}&\in \{0,1\}  &   \forall i,j\in \mathcal {I} \end{aligned}$$

The TSP’s objective is to minimize the distance between coupled NICU locations. Constraint (B2) states that all locations must be visited. Constraint (B3) ensures that each location that is visited is also left. Constraint (B4) eliminates sub-tours by ensuring that each potential sub-tour is left.

Since the model is analyzed with different combinations of participating locations, the shortest route is calculated for each variation. The tour can be followed clockwise or counterclockwise. Figure 13 shows one of these two directions. The reason this route is chosen instead of the other option is that this route minimizes the distances traveled from the larger NICUs (i.e., Amsterdam, Rotterdam, and Utrecht). Further analyses, not reported, showed the reverse route for the configurations in which all locations are included did not provide improved performance results.

The chaining cross-training policy can be followed clockwise or counterclockwise. No significant differences in performance are observed in outcomes in the percentage of rejected patients and the percentage of transported patients in initial experiments for clockwise and counter-clockwise chaining. Therefore, we will use the chain direction that minimises the travel distance from the larger NICU locations (Amsterdam, Rotterdam, Utrecht), as this setting has a slightly lower percentage of transported nurses.
